# Update on Malignancy in Myositis—Well-Established Association with Unmet Needs

**DOI:** 10.3390/biom12010111

**Published:** 2022-01-11

**Authors:** Aleksandra H. Opinc, Joanna S. Makowska

**Affiliations:** Department of Rheumatology, Medical University of Lodz, Żeromskiego 113, 90-549 Lodz, Poland; aleksandra.opinc@umed.lodz.pl

**Keywords:** cancer-associated myositis, myositis, idiopathic inflammatory myopathy, cancer, neoplasm, malignancy

## Abstract

Idiopathic inflammatory myopathies are a group of rare connective tissue diseases with a well-documented association with malignancy. The mechanisms underlying the increased risk of neoplasms in the course of myositis are not fully understood. The Pubmed database has been thoroughly screened for articles concerning cancer-associated myositis (CAM). The article summarizes the current state of knowledge on the epidemiology and pathogenesis of CAM. Furthermore, it analyses potential risk and protective factors for developing CAM, with particular emphasis on the association with distinct serological profiles. The review summarizes recommendations proposed so far for the management of CAM and presents a novel scheme for cancer screening proposed by the authors. Moreover, promising areas requiring further research were indicated.

## 1. Introduction

Idiopathic inflammatory myopathies (IIM) are a group of rare connective tissue diseases. According to the literature, the yearly incidence of IIM ranges from 1.16 to 19/1,000,000 and the disease prevalence was estimated as 2.4–33.8/100,000 [1]. Several subtypes can be distinguished such as dermatomyositis, polymyositis, inclusion body myositis, antisynthetase syndrome and immune-mediated necrotizing myopathy [2]. In some patients, further subclassifications are useful for example cancer-associated myositis to highlight the concomitance with malignancy. Typical symptoms of IIM include muscle weakness. In the course of IIM, internal organs may be also involved, leading to multisymptomatic clinical presentation [3].

Cancers remain the second cause of death worldwide, following deaths caused by cardiovascular events. Although in recent years, a decreasing trend is observed in cancer mortality, global incidence rates are constantly increasing [4]. According to the Global Burden of Disease Study from 2015, the most prevalent types of cancer were prostate, lung, colorectal and breast tumors [5].

Although the association between cancer and myositis was documented for the first time over 100 years ago [6,7], the mechanisms underlying this phenomenon remain not fully understood. Possible life-threatening consequences of concomitant malignancy impose an obligation to actively screen for cancers. The aim of the study was to summarize current knowledge about cancer-associated myositis (CAM) and its epidemiology, pathogenetic background, risk factors and clinical course. Despite the lack of clear guidelines for the management of CAM, outlines proposed so far have been described.

## 2. Epidemiology

Diagnosis of cancer-associated myositis (CAM) can be posed if malignancy occurs within three years of the diagnosis of inflammatory myopathy, including both the period following and proceeding the onset of myositis [8,9]. The definition of CAM has been based on numerous epidemiological observations [10,11,12,13,14]. The newest population-based studies confirm that the majority of malignancies occur in the temporal association with the onset of myopathy. Most of the cancer cases emerge within a year of IIM diagnosis, and the risk of malignancy decreases over time [15,16,17]. The temporal coincidence and the disappearance of muscle symptoms after tumor removal enabled us to conclude that CAM may be a paraneoplastic syndrome [9]. However, it needs to be underlined that malignancy treatment does not always lead to the diminishing of IIM symptoms, as idiopathic inflammatory myopathies and cancer frequently follow an independent course. 

According to the EuroMyositis registry, malignancies occurred at any time in 13% of patients with myositis, with most of them being diagnosed in close temporal association with the onset of myopathy [18]. In most cases, cancer develops simultaneously with the onset of myopathy or within the first year of diagnosis, but the risk, although gradually decreasing, remains elevated for several years [8,19]. SIR of malignancy has been estimated as 17.29 in the first year following the diagnosis of dermatomyositis, yet only 1.37 five years post-diagnosis [15]. In the retrospective study performed by András et al. on a Hungarian cohort of 450 patients with IIM, over 83% of patients with CAM developed cancer within the first year after diagnosis [20]. This remains in line with data from the national registries of Sweden, Denmark and Finland, in which the onset of myositis was associated with the highest risk of malignancy [21]. The course of myositis was reported to be more severe if the cancer was diagnosed concurrently than when the entities emerged at longer time intervals [22].

The highest risk of malignancy is observed in the course of dermatomyositis. Increased risk of neoplasm is also observed in individuals with polymyositis, yet it is not as high as in dermatomyositis (standardized incidence ratios of 3.8–7.7 in DM—dermatomyositis vs. 1.7–2.2 in PM—polymyositis patients) [23]. According to the meta-analysis of five studies on the total group of 4538 patients with myositis, the overall relative risk for developing malignancy was 4.66 (95% CI 3.32–6.52) for patients with dermatomyositis and 1.75 (95% CI 1.37–2.25) for patients with polymyositis [15]. Surprisingly high incidences of CAM were observed in the Scandinavian region, as according to pooled data from national registries, patients with cancer-associated dermatomyositis accounted for 32% of all DM patients, and cancer-associated polymyositis was diagnosed in approximately 15% of PM patients [21]. Cancers were also frequently observed in patients with clinically amyopathic dermatomyositis (patients with skin lesions typical for DM, without apparent clinical symptoms of muscle involvement) [24]. Noteworthy, in patients with antisynthetase syndrome, risk of malignancy seems to be noticeably lower than in remaining types of myositis [8]. Incidence of cancer in the course of inclusion body myositis seems to be inconsistent [8]. In the study by Buchbinder et al. on a group of 537 patients with biopsy-proved idiopathic inflammatory myopathy, increased risk of malignancy was observed in the subgroup of patients with IBM (standardized incidence ratio of 2.4) [25]. In the study based on the South Australian myositis database with 373 registered patients, malignancies occurred in 11.3% of patients with IBM [26]. Although SIR of malignancy was 1.37 in patients with this subtype, it remained not statistically significant [26]. On the contrary, data from the Norwegian patient administrative databases indicated increased cancer risk in DM and PM but not IBM (SIR = 0.9) [27]. Compared to DM and PM, available data on malignancy in IBM is limited. Further studies are expected to reliably assess the risk of cancer in this subtype of IIM.

Neoplastic diseases were found to occur more frequently in elderly patients [12]. According to the meta-analysis, the standardized incidence ratio was estimated as 2.79 for patients aged 15–44 and 3.13 for patients over 45 years old [15]. Similarly, in the study by András et al., patients with CAM were statistically older than patients with isolated myositis (respectively 56.60 ± 12.79 vs. 38.88 ± 10.88 years) [20]. The risk of cancer was higher in men with DM (SIR of 5.29 in men vs. 4.56 in women) as compared to women in PM (SIR of 1.62 in men vs. 2.02 in women) [15].

The above data prove that cancer risk is not uniformly increased for all patients with IIM. The highest risk exists around the time of IIM diagnosis. Individual factors, such as the patient’s age and the subtypes of the disease, also impact the risk.

## 3. Pathogenesis

The frequent coexistence of neoplasms in the course of inflammatory myopathies prompted researchers to search for the cause at the genetic and molecular levels. 

Genetic factors are suspected to underlie CAM. In the Caucasian population, HLADQA1*0301 was associated with the presence of anti-p155/140 autoantibodies (anti-TIF1-γ), which are considered as a risk factor for CAM [28].

It is now believed that mutations or abnormal expression of autoantigens genes in a neoplasm are able to induce cross-reactivity against own proteins, inducing paraneoplastic myositis [9]. Casciola-Rosen et al. have demonstrated significantly higher expression of antigens such as Mi-2 or Jo-1 in muscle samples from myositis patients, with the most prominent expression observed in damaged regenerating fibers compared to healthy individuals [29]. Noteworthy, those antigens were found to be overexpressed also in myositis-associated adenocarcinomas of breast and lung, yet not in the corresponding healthy tissues [29]. Similarly, a case report of a patient with dermatomyositis and endometrial cancer with high expression of TIF1-γ in tumor cells was described [30]. That observation led to the hypothesis of cross-reactivity between immune response against cancer and regenerating muscles. According to this theory, antigens exposed by the tumor initiate an immune response directed against the neoplasm. If muscle injury occurs due to, for example, infection, trauma or toxin exposure (“second hit”), regenerating muscles start to express myositis-specific antigens, becoming a target for cross-over immune reaction, which leads to the outbreak of inflammatory myopathy [19,29,31]. Many studies confirm the improvement in the clinical presentation of myositis post-tumor resection or treatment, which possibly corresponds to a decrease in autoantigen burden [19]. However, in some cases, the symptoms of myositis return even without the cancer recurrence, supporting the hypothesis that the immune response, initially induced by the tumor, becomes independent and self-propelling against muscular and cutaneous antigens [9].

Interestingly, Hengstman et al. demonstrated that patients with CAM generate immune responses towards different epitopes of myositis-related autoantigens, such as Mi-2, than patients with isolated myositis [32].

Furthermore, in the samples of muscle tissue derived from patients with early-stage colon cancer but without apparent myositis, histopathologic features of myopathy, resembling the presentation observed in CAM, were detected [33]. Shared features included the presence of internally nucleated fibers and immunohistochemical staining for markers of regeneration, with high expression of neural cell adhesion molecule [33]. These observations support the hypothesis of cross-reactivity between the neoplasm and regenerating myofibers.

The overexpression of antigens by tumor cells leads to abnormal processing or cleavage of the tumor antigen with subsequent generation of epitopes not previously encountered by the immune system and in consequence triggers an immune response. It remains in line with the previously described observation by Casciola-Rosen et al. [29]. One of the mechanisms in which the tumor escapes from the suppressive control of the immune system is the loss of heterozygosity (LOH). In the study by Pinal-Fernandez et al. whole-exome sequencing analysis of TIF1 genes was performed in seven anti-TIF1-γ-positive CAM patients to search for somatic mutations and LOH in tumors. One somatic mutation and five cases of LOH were identified in one or more of the four TIF- γ genes. On the contrary, in anti-TIF1-γ-negative patients, only a single case of LOH was found [34]. Moreover, the authors demonstrated more prominent TIF1-γ staining in tumors and muscles from anti-TIF1-γ-positive patients as compared to anti-TIF1-γ -negative subgroup [34]. TIF1-γ staining was also clearly visible in skin samples [30]. In the shield of those observations, it has been concluded that immune response with production of anti-TIF1-γ antibodies is initiated by mutations in TIF1 genes occurring in tumors and directed against mutated neoantigen. Neoplasm escapes the immune system by loss of heterozygosity, which leads to a progression of the tumor and redirecting the target of the immune response towards other tissues with high expression of TIF1 antigens—myofibers and skin cells. Damage and regeneration of the myofibers promote overexpression of TIF1 protein, leading to enhancement of immune reaction [34,35]. Tumour itself can also contribute to the increase of antigen expression by inflammatory-dependent muscle wasting or cachexia [35].

Checkpoint inhibitor pathway may contribute to the development of cancer-associated dermatomyositis. In the study by Chen et al., levels of soluble programmed death ligand 1 (sPD-L1) were measured and compared between three subgroups: patients with DM without cancer, patients with CAM and healthy controls [36]. As PD-1/PD-L1 pathway activation suppresses immune responses, higher concentrations may indicate a tumors’ ability to effectively escape from the control of the immune system. According to Chen et al., patients with myositis both with and without malignancy presented higher levels of sPD-L1 than healthy individuals [36]. Noteworthy, the sPD-L1 levels in newly-onset CAM exceeded significantly levels observed in stable CAM and decreased after tumor treatment [36]. So far, the role of the PD-1/PD-L1 pathway in IIM has only been assessed in a single study. Further studies on larger groups are necessary to assess the significance of checkpoint inhibitors in the development of CAM.

Finally, the involvement of tumor-infiltrating lymphocytes has been suspected to contribute to CAM [35].

**Figure 1:**
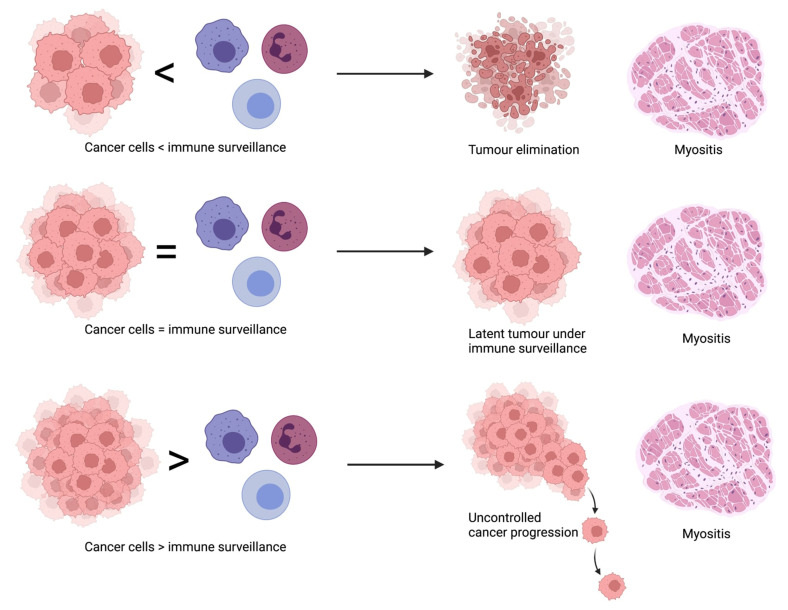
Courses of malignancy in myositis.

The above observations led to the formulation of an incredibly appealing theory that an outbreak of a fully symptomatic myositis may prove the temporal victory of immune surveillance over a developing tumor [19,35]. It is worth emphasizing that in the vast majority of patients with myositis, the disease is not accompanied by cancer, suggesting a highly efficient surveillance mechanism, which however carries a cost of myositis development as its side effect. Three possible courses of malignancy in myositis have been proposed—elimination, in which the immune system cease the growing tumor yet induces inflammatory myopathy; equilibrium, when in the patient with clinically apparent myositis tumor cells persist in latency under the surveillance of the immune system with a risk of progression if the control is lost; and escape, when a patient with myositis tumor overcomes the immune surveillance by, for example, LOH, preceding the uncontrolled expansion of the tumor [35]. A summary of the proposed theory has been presented in Figure 1.

## 4. Types of Cancers Associated with Myositis

In the course of idiopathic inflammatory myopathies, different types of cancers have been observed. Similar to the neoplasms noted in the general population, adenocarcinoma seems to be the most prevalent tumor type in patients with myositis, as it accounts for approximately 70% of malignancies in CAM [21]. Lung, gastrointestinal, ovarian, breast, cervical, bladder, uterine, pancreatic and prostatic tumors, as well as Hodgkin’s lymphomas, are listed among the most frequent neoplasms associated with IIM [8,19].

The prevalence of cancers may depend on the subtype of myositis, as specific malignancies occur more frequently in patients with DM while other cancers are predominantly diagnosed in patients with PM. According to data from Scandinavian registries, the most frequently detected tumors in patients with DM included ovarian, pancreatic, stomach and colorectal cancer as well as non-Hodgkin’s lymphoma. The relative risk of hematologic neoplasms was found to be higher in PM than in DM, with non-Hodgkin’s lymphoma being the most prevalent malignancy, preceding lung and bladder cancers [21]. Separately analyzing different types of hematologic malignancies, B-cell lymphomas were found to be the predominant ones in CAM patients [37]. According to data from the nationwide cohort study of 1012 patients with DM and 643 patients with PM from the Taiwanese National Health Insurance Research Database, patients with DM nasopharyngeal, lung/mediastinal and bone cancers as well as lymphoma/leukemia were the most commonly diagnosed, while PM patients had the highest risk of bone, cerebral and nasopharyngeal cancers and melanomas [38].

Interestingly, significant discrepancies have been observed in the types of malignancies occurring in patients of various races and ethnicities [8]. Observed differences remain consistent with distinct profiles of malignancies observed in general populations inhabiting different parts of the world. Patients from Western countries with CAM usually develop ovarian, lung or gastrointestinal adenocarcinomas, while in Asia and Northern Africa, nasopharyngeal carcinoma is the most prevalent [23]. The risk for nasopharyngeal carcinoma was reported to be 66-fold higher for individuals with myositis than for the general population [39]. In Chinese patients with DM, nasopharyngeal, lung and breast cancers were most commonly diagnosed, while in PM patients, breast, uterine, cervical and lung cancers were the most frequent [39,40]. Cases of colon, stomach and hepatobiliary tract cancers were also reported as commonly occurring in the Asian population of patients with myositis [40,41]. Hematologic malignancies accounted for a quarter, while prostate cancer for 20% of all neoplasms observed in CAM patients from the south Australian cohort [26].

Lung and ovarian tumors are one of the most prevalent malignancies worldwide, which is also reflected in the myositis population. Both of these tumors are characterized by no apparent symptoms at the onset of the disease, which lead to delay in diagnosis and in consequence, to high mortality. Moreover, no cost-effective screening tests are available for the early detection of ovarian cancer, and the use of low-dose CT of the chest for lung cancer detection is not widespread. Ovarian cancer, one of the most prevalent tumors in European patients with CAM, typically derives from the epithelium and usually develops within the first year of myositis [42]. Unfortunately, diagnosis of the concomitant neoplastic process is frequently stated at the late stage of the disease—stage III or IV, which significantly worsens the prognosis [42].

Lung cancer is one of the most common malignancies worldwide, which is also reflected in the myositis population. In patients with DM and PM, subtypes such as small cell carcinoma, squamous cell carcinoma and adenocarcinoma of the lung were reported as the most prevalent [43].

Due to the variety of detected neoplasms, screening for malignancy should be comprehensive. While planning the range of screening tests, it seems justified to consider racial differences and the resulting susceptibility to specific cancers, as well as factors related to IIM itself, such as the subtype of the disease.

## 5. Risk Factors for Malignancy in Myositis

Many researchers have searched for factors that could predict an increased risk of cancer in patients with myositis. Data from various publications remains fairly consistent.

### 5.1. Clinical Factors Associated with CAM

#### 5.1.1. Demographical Data

One of the factors strongly associated with the risk of malignancy is older age at the onset of myositis [12,37,38,44,45,46,47,48,49,50]. Slightly different results were obtained by Stockton et al., who demonstrated that age above 45 was associated with increased risk of malignancy only in DM patients, while in the PM group, tumors occurred more frequently in young or middle-aged patients [51]. The data remain less conclusive about the risk of cancer associated with the patient’s gender, but most of the authors conclude that the male gender increases the risk of cancer in idiopathic inflammatory myopathies (IIM) [11,26,44,45,46,50]. Independent meta-analyses also demonstrated that the male sex is associated with a higher risk of co-occurring malignancy, while the risk in women with PM could be comparable to the risk observed in the general population [48,49,52]. According to a meta-analysis of 69 studies by Oldroyd et al., older age at the IIM onset as well as male sex were confirmed to be major risk factors of malignancy in IIM [53].

#### 5.1.2. Muscle Involvement

Patients with severe muscle involvement are also considered to have a higher risk of malignancy. Cancers were found to occur more frequently in patients with prominent skeletal muscle weakness as well as in patients with the involvement of the distal skeletal muscles of the limbs, skeletal respiratory muscles and esophageal muscles [12,14,38,45,47,48]. Dysphagia, which may be caused by the involvement of both skeletal muscles of the oropharynx and esophagus as well as smooth esophageal muscles [54], has been confirmed to be a significant risk factor in the meta-analysis by Oldroyd et al. [53]. Rapid onset of myopathy should also encourage greater oncological vigilance [44,47,49]. Infiltrating inflammatory cells without perifascicular atrophy, observed in muscle biopsies from patients with DM, could also serve as a biomarker of increased cancer risk [55].

#### 5.1.3. Cutaneous Involvement

Malignancy was reported to be associated with severe cutaneous lesions. The presence of several specific skin pathologies such as cutaneous ulceration, skin necrosis and leukocytoclastic vasculitis seem to indicate high malignancy risk [12,44,47,49,56,57]. Periungual erythema, violaceous and heliotrope rash, Gottron’s papules and essential derangement of nailfold capillaries were also reported in CAM patients [44,57,58].

#### 5.1.4. Biochemical and Serological Tests

Laboratory findings could also shed light on the probability of malignancy. Lower concentrations of creatine kinase and lactate dehydrogenase were found to be associated with an increased risk of malignancy [53]. Highly elevated markers of inflammation such as C-reactive protein and erythrocyte sedimentation rate, unusual for isolated IIM, were associated with concomitant cancers [41,59]. Low levels of C4 were found to be an independent risk factors for cancer [44].

### 5.2. Serological Profiles Associated with CAM

Certain serological profiles are predominant in patients with CAM. Higher risk of malignancy in the course of IIM have been reported in patients with antibodies targeting transcriptional intermediary factor 1-γ (TIF1-γ), nuclear matrix protein-2 (NXP-2), 3-hydroxy-3-methylglutaryl-coenzyme A reductase (HMGCR) and small ubiquitin-like modifier 1-activating enzyme (SAE), as well as in seronegative patients [8,9]. Among them, anti-TIF1-γ antibodies are of fundamental importance as the most strongly associated with cancer risk.

#### 5.2.1. Anti-TIF1-γ Antibodies

**Figure 2:**
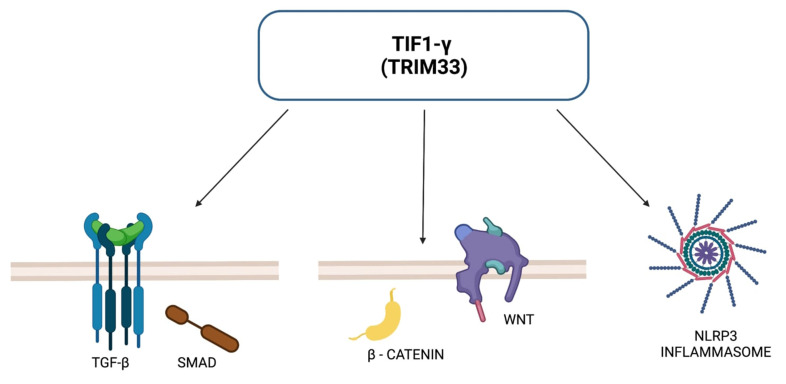
TRIM33-dependent pathways for tumor suppression.

Antibodies against TIF1-γ are specific for DM and are not observed in other subtypes of IIM. Association with anti-TIF1-γ antibodies and CAM have been well established by numerous studies. TIF1-γ, known also as TRIM33, is a member of tripartite motif family proteins, which exert multiple roles within our cells. TRIM33 has been demonstrated to participate in DNA repair, transcript elongation, cell mitosis and differentiation, hematopoiesis and embryonic development. TIF1-γ regulates the TGF-β/Smad signaling pathway, the activation of which is fundamental for tumor growth and metastasis suppression. TIF1-γ, similarly to the other proteins from the TRIM family, has ubiquitinating ligase activity. It is able to inhibit the TGF-β/Smad signaling pathway by monoubiquitinating of Smad4 protein or by acting as a cofactor for the p-Smad2/3 complex, inhibiting the formation of the Smad2/3/4 complex [60,61]. It has been shown that in glioblastomas, TIF1-γ can regulate the Wnt/β-catenin signaling pathway—which influences tumor growth and metastasis—through ubiquitination of β-catenin degradation, leading to inhibition of cell proliferation and tumor formation [60,61]. Another potential mechanism of action is the pathway associated with NLRP3. It is known that activation of the NLRP3 inflammasome complex requires the help of TIF1-γ. Dysregulated expression of NLRP3 inflammasome was found in squamous cell carcinoma of the head and neck, hepatocellular carcinoma and colorectal cancer; furthermore, it may be involved in resistance to radiotherapy and chemotherapy in certain malignancies [60]. TIF1-γ acts as a suppressor in tumors such as non-small cell lung cancer, breast cancer, glioblastoma and clear cell kidney cancer. However, for other cancers—lymphoblastic leukemia B, pancreatic cancer and cervical cancer—TIF1-γ may act as a tumor promoter [60,61]. Figure 2 presents ways in which TRIM33 may act to inhibit tumor growth.

As compared to IIM patients without cancer, in adult individuals with CAM the prevalence of anti-TIF1-γ antibodies was found to be significantly higher [62]. In the Chinese cohort described by Li et al., 34 out of 477 patients with IIM developed malignancy. Although anti-TIF1-γ was detected in 14.3% of the study group, patients with reactivity against TIF1-γ accounted for 61.8% of the group with detected malignancy [63]. Similar results were reported in another Chinese cohort, where the prevalence of anti-TIF1-γ antibodies was significantly higher in CAM patients (46.9%) than in myositis patients without concomitant malignancy (14.8%) [62]. Aussy et al. demonstrated that only the anti-IgG2 isotype of anti-TIF1-γ antibodies correlated with concomitant cancer in the course of DM. High fluorescence intensity of anti-TIF1-γ IgG2 could serve as a novel biomarker of malignancy as it had a 100% positive predictive value of neoplasm in myositis patients [64]. The probability of coexisting neoplasm is higher if the titer of anti-TIF1-γ antibodies increases after treatment [65]. Considering DM patients with malignancy, cancers in anti-TIF1-γ-positive patients were usually more advanced than in patients without those antibodies [66]. The majority of tumors in the course of DM with TIF1-γ reactivity were detected either simultaneously or promptly after the diagnosis of myopathy [9]. Although anti-TIF1-γ are also frequent in juvenile dermatomyositis, in the pediatric population, they seem not to correlate with increased risk of malignancy [8].

Interestingly, in the recent cross-sectional study the presence of antibodies associated with systemic autoimmune rheumatic diseases have been confirmed in 22 out of 152 patients with breast cancer without concomitant rheumatic diseases. Antibodies against TRIM33 were identified in two patients, following antibodies against Ro-52 in nine cases, antibodies against Ro-60 in 6 and anti-Su antibodies in four patients [67].

#### 5.2.2. Anti-NXP-2 Antibodies

The presence of anti-NXP-2 antibodies was suggested as a possible risk factor for malignancies [24], most of all for the formation of solid tumors [9]. Antibodies against NXP-2 are predominantly observed in patients with dermatomyositis and juvenile dermatomyositis; however, the case of anti-NXP-2-positive immune-mediated necrotizing myopathy was also reported [68,69,70].

NXP-2, also known as MORC3 (microrchidia 3), is a nuclear protein belonging to a nuclear matrix protein family. It participates in DNA repair, chromatin remodeling and epigenetic regulation, as well as promotes the activation of p53 and co-acts in cellular proliferation. Furthermore, it is involved in bone remodeling and calcium homeostasis [71].

In a study by Ichimura et al., cancer was diagnosed in 37.5% of patients with anti-NXP-2 antibodies, yet the reliability of this observation is limited by the rarity of this serological profile in the study group (only eight patients with anti-NXP-2) [72]. Fiorentino et al. performed an analysis of two separate cohorts from Standford and John Hopkins Universities, revealing that 83% of the patients with cancer-associated myositis presented reactivity against TIF1-γ or NXP-2. Taking into account the total number of patients from both cohorts, tumors were found in 24.32% of patients with anti-NXP2 antibodies and 18.3% of patients with anti-TIF1-γ. Antibodies to NXP-2 were found to be associated with a high risk of malignancy with an OR of 5.8 yet only in male patients with IIM [73]. However, data from the literature remain not fully consistent. According to the meta-analysis by Oldroyd et al., the risk ratio of anti-NXP-2 was non-significant [53]. In the Chinese cohort, only one patient out of eight with anty-NXP-2-positivity developed malignancy [62]. Similarly, in the recent meta-analysis of 20 studies with a total number of 3064 patients with myositis, the association of anti-NXP-2 antibodies with increased risk of malignancy was not confirmed [74]. It is noteworthy that the meta-analysis included patients from various nationalities, which may suggest that the association of increased cancer risk with NXP-2 antibodies is significant only in some ethnic groups and diminishes in a multi-ethnic society.

#### 5.2.3. Anti-SAE Antibodies

Antibodies against SAE are rare and specific for DM and its juvenile form [75,76,77]. Anti-SAE antibodies are considered a predictive factor for concomitant malignancy, yet compared to previous antibodies, less data are available to support this association. According to the literature, anti-SAE-positive patients developed mostly adenocarcinomas of the cervix, lung or gastrointestinal track [9].

In the Chinese cohort of 627 patients with IIM, a statistically significant association of anti-TIF1-γ, anti-NXP2 and anti-SAE1 antibodies with a high risk of cancer were confirmed [49]. Out of 72 patients with malignancy, 34 were positive for anti-TIF-1-γ (38.2% out of all anti-TIF1-γ-positive patients), 3 were anti-NXP-2-positive (7.14% out of all 42 patients with anti-NXP-2) and 4 were positive for anti-SAE (30.77% out of 14 anti-SAE-positive patients). The presence of specific antibodies did not predict the type of malignancy [16].

#### 5.2.4. Remaining Serological Profiles

Antibodies against HMGCR are highly specific for immune-mediated necrotizing myopathy [78]. Data on the association of anti-HMGCR antibodies with an increased risk of cancer remain inconsistent [9]. In some of the studies, a higher incidence of neoplasm was reported in anti-HMGCR-positive patients as compared to the general population, yet it was not confirmed in other studies [79,80]. According to the meta-analysis, the association of this serological profile with the risk of malignancy remained non-significant [53]. The majority of malignancies in the course of necrotizing autoimmune myopathy with antibodies against HMGCR occurred within the first three years after the diagnosis of myositis [9]. Cases of cancers in patients with antibodies targeting Jo-1 and PL-12 were reported [9], yet in other studies, the presence of antisynthtease syndrome was a protective factor [8]. Indeed, the meta-analysis revealed a reduced risk of malignancy in patients with antisynthetase antibodies compared to the remaining IIM patients [53]. So far, there are too little data to attribute an increased risk of cancer to the antisynthetase syndrome [35]. The association of sporadic inclusion body myositis with an increased incidence of neoplastic diseases remains inconsistent [35]. The presence of anti-SRP, anti-MDA5 or anti-Mi-2 antibodies was found to be non-significant in relation to the risk of cancer [53]. The risk of CAM was found to be increased also in seronegative patients with IIM, in whom no myositis-specific or myositis-associated antibodies were detected by routine tests [81].

## 6. Protective Factors

Several clinical symptoms have been associated with a lower risk of cancer. Many researchers found that interstitial lung disease in the course of IIM decreases the risk of malignancy, but the underlying mechanism remains unknown [12,20,38,45,46]. Other symptoms from the spectrum of an antisynthetase syndrome such as arthritis/arthralgia, fever and the Raynaud phenomenon also seem to diminish the risk of tumors [14,45]. On the contrary, the asymmetric Raynaud phenomenon was associated with a higher incidence of malignancy [20]. Cardiac involvement has been also considered a cancer-protective factor [14]. Fardet et al. demonstrated in the retrospective analysis the negative association of lymphopenia and increased probability of malignancy [44].

## 7. Management of Cancer-associated Myositis

Although the association of IIM with cancer has been well-established for years, no clear guidelines have been published so far on the management of co-occurring malignancy. As versatile types of neoplasm can develop in patients with myositis, screening for latent malignancy should be comprehensive.

Recently, an interesting expert opinion on a proposed screening scheme has been published. Moghadam-Kia et al. proposed to stratify the risk of malignancy depending on the subtype of IIM, clinical and serological factors [8]. Patients with dermatomyositis with at least two clinical risk factors (such as older age, male gender, dysphagia, cutaneous necrosis or ulcerations, vasculitis, rapid onset of IIM, refractory course of IIM, high concentrations of muscle enzymes and inflammatory markers) and no protective factors (ILD, inflammatory arthropathy, Raynaud phenomenon), as well as patients with anti-TIF1-γ and anti-NXP-2 antibodies were considered high-risk groups. Seronegative patients, individuals with anti-SAE and anti-HMGCR antibodies were included into subgroups with moderate risk, similarly as clinically amyopathic or polymyositis patients with one or two clinical risk factors in the presence of any of the protective factors. The remaining patients were classified as probably having a low risk of malignant disease [8]. The authors recommended adjusting the scope of screening tests to the degree of risk with basic screening for those at low risk, enhanced in the intermediate-risk group and comprehensive evaluation in patients with high risk. For patients with the lowest risk, it seems advisable to perform a blood test, chest X-ray and screening tests recommended in the general population depending on age including colonoscopy, cervical cytology, mammography and prostate-specific antigen. If the risk is moderate, additional examinations should be considered including computed tomography of the chest or abdomen and pelvis, pelvic or transvaginal ultrasonography to exclude ovarian cancer and testicular ultrasonography in men below 50. For patients with high risk, PET examination should be taken into account. Although no clear recommendations are available as for the frequency of cancer screening, researchers advised to perform it at least at the onset of myositis in patients with lower risk and once a year for the first three years after diagnosis in patients with high risk [8]. Moreover, the authors of the proposed scheme suggested assessing tumor markers in patients with at least a moderate risk [8]. In the prospective study by Amoura et al., on 102 patients with myositis, the elevation of CA125 on the onset of myositis was associated with an increased risk of malignancy [82]. A similar tendency, yet statistically non-significant, was observed for CA19-9 [82]. The usefulness of carcinoembryonic antigen and CA 15-3 in the detection of neoplasms in patients with myositis has not been confirmed [82]. On the other hand, in the single case-control study with 14 patients with DM, out of which four had ovarian cancer, the CA125 marker had only 50% sensitivity for this type of neoplasm [83]. Considering the limited number of participants in the above study, minimally invasive nature of the study and the high-risk and insidious course of ovarian cancer, it seems highly advisable to evaluate serum CA125 concentration in women with myositis.

In the recently published review, nine studies on cancer screening in IIM were analyzed [53]. Out of versatile screening methods, CT scanning of the thorax, abdomen and pelvis was found to be especially useful [53]. According to Leatham et al., CT screening enabled to reveal 38% (6/17) of subclinical cancers in IIM, followed by mammography, which detected 18% (3/17) [84]. Similarly, in a study by Sparsa et al., while analyzing the screening methods separately, chest and abdominopelvic CT was the most effective in the detection of asymptomatic neoplasms [85]. Notably, the addition of ^18^F-FDG PET/CT to diagnostics did not increase the number of neoplasms found and, according to some authors, increased the frequency of unnecessary biopsies [53,86,87].

Adjustment of the scope of performed tests to the ethnic origin of the patients is recommended due to the observed differences in the incidence of particular neoplasms [23]. Special vigilance is needed in order not to overlook malignancies such as ovarian, pancreatic or lung cancers as well as lymphomas [11].

**Figure 3:**
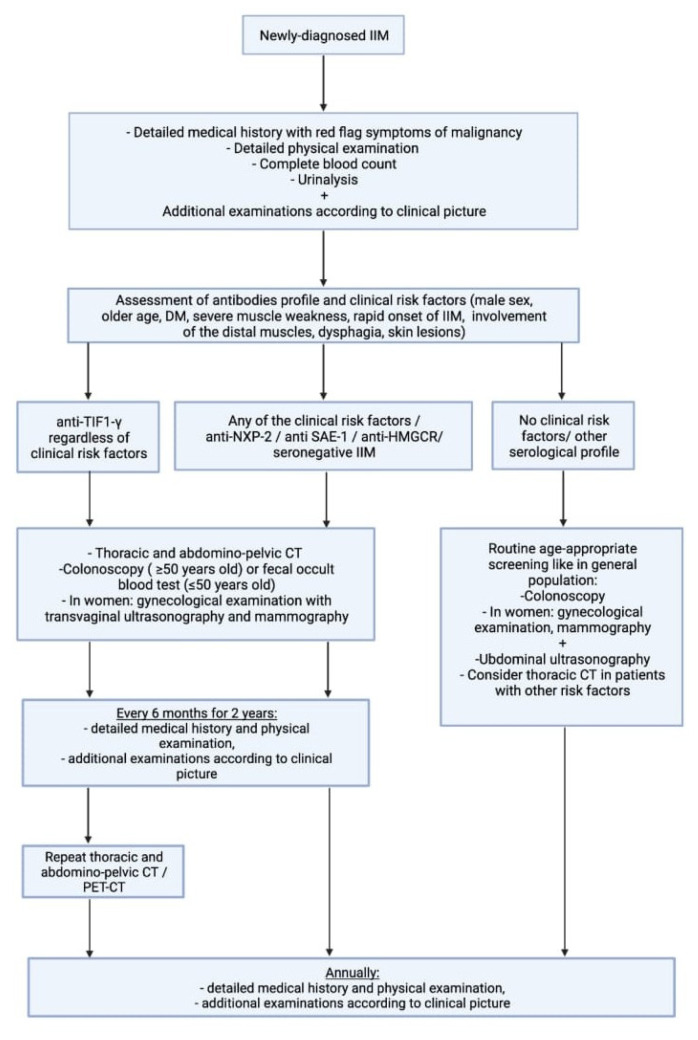
Proposed scheme for cancer screening. IIM-idiopathic inflammatory myopathy, PET-positron emission tomography, CT-computed tomography.

As evidence on the increased risk of neoplasm associated with antibodies other than anti-TIF1-γ remains unclear, we proposed an alternative scheme for cancer screening (Figure 3). Due to the high risk of cancer not only at the onset of myositis but also within the first few years, we propose intensive screening at the beginning with annual re-evaluation even in patients without risk factors. In patients with anti-TIF1-γ antibodies, as the subgroup with the highest risk of malignancy, imaging examination tailored to the regional availability (PET or CT) should be considered annually.

## 8. Prognosis

The survival rate is worse in patients with CAM as compared to patients with primary myositis [18]. Cases of improvement or remission of muscular symptoms were reported after tumor treatment, whereas recurrence of malignancy could trigger an exacerbation of myopathy [12,23]. These observations prove that cancer and myositis are closely bounded and not merely coexisting.

## 9. Unmet Needs

As there are no evidence-based medicine recommendations, treatment and management of patients with CAM should be so far based on expert opinion. Clear guidelines on the diagnosis and management of patients with CAM are highly anticipated. Patients with CAM require dual care—treatment of malignancy and myositis. Such treatment should include intensive cooperation between rheumatologists, oncologists, radiologists and frequently other specialties. Studies on the risk of malignancy in different ethnic groups could shed novel light on risk factors, as certain associations, e.g., the impact of autoantibodies on cancer risk, could be dependent on demographical data. Further studies on larger groups are also needed to estimate the risk associated with less prevalent serological profiles. Soluble PDL-1 level emerges as a promising biomarker of CAM, indicating an appealing path for future studies.

## Data Availability

Not applicable.
